# Dorsal proximal interphalangeal joint tenderness is associated with prolonged postoperative pain after A1 pulley release for trigger fingers

**DOI:** 10.1186/s12891-023-06130-5

**Published:** 2023-01-07

**Authors:** Yuwarat Monteerarat, Pimolpan Misen, Panai Laohaprasitiporn, Pattarawat Wongsaengaroonsri, Nittaya Lektrakul, Torpon Vathana

**Affiliations:** 1 Department of Orthopaedic Surgery, Faculty of Medicine Siriraj Hospital, Mahidol University, Bangkok, Thailand; 2 Department of Radiology, Faculty of Medicine Siriraj Hospital, Mahidol University, Bangkok, Thailand

**Keywords:** A1 pulley release, Postoperative pain, Proximal interphalangeal (PIP) joint tenderness, Trigger fingers

## Abstract

**Background:**

In some trigger finger patients, tenderness is found in the dorsal proximal interphalangeal (PIP) joint. The etiology and prevalence of this condition are unclear. Furthermore, surgical outcomes for trigger fingers with coexisting dorsal PIP tenderness have not been reported. This study (1) determined the prevalence and risk factors for PIP joint tenderness in trigger fingers and (2) compared postoperative outcomes for trigger fingers with and without joint tenderness.

**Methods:**

This prospective cohort study was conducted between August 2018 and March 2020. We enrolled 190 patients diagnosed with single-digit trigger fingers undergoing open A1 pulley release. The incidence, demographic data, and surgical outcomes of patients with dorsal PIP tenderness were investigated. Factors associated with tenderness were analyzed, including patient occupation, finger involvement, trigger finger grading, duration of symptoms, previous corticosteroid injections, and presence of diabetes mellitus. A numeric pain scale, a patient-specific functional scale, and the range of motion were evaluated preoperatively and 1, 2, and 6 weeks after surgery, with telephone follow-ups at 3 and 6 months.

**Results:**

Of 190 patients, 46.8% had tenderness of the dorsal PIP joint. Patients with joint tenderness had significantly more overall postoperative pain for up to 6 weeks and reported residual minor pain for up to 3 months. The functional scale and range of motion of the 2 groups did not differ during follow-up. The only risk factor observed was the occupation of the patients.

**Conclusion:**

Dorsal PIP tenderness is more common in trigger fingers than previously thought. It is also associated with higher and prolonged levels of postoperative pain after A1 pulley release. Therefore, patients with pre-existing PIP tenderness should be informed about the possibility of sustaining residual minor pain for up to 3 months after surgery.

**Level of Evidence:**

II

## Introduction

Trigger finger, or stenosing flexor tenosynovitis, is one of the most common hand problems. The condition is characterized by painful locking or catching when flexing or extending the finger or by thumb motion, followed by a disability of hand function [1]. Pathology comprises thickening of the A1 pulley, an inflammatory nodule, and local thickening of the flexor tendon around the A1 pulley [1, 2]. Although the primary pathology is localized at the metacarpophalangeal joint, some patients complain of additional pain at the proximal interphalangeal (PIP) joint, especially during active joint movement. The pain presents mainly in the dorsal PIP joint, concomitant with joint swelling and tenderness on palpation.

In 2018, Kim and colleagues first reported the clinical significance of PIP joint pain in patients with trigger fingers. Their research found 24% of patients with trigger fingers had preoperative PIP joint pain, but only 13% had PIP tenderness. Furthermore, 40% of their patients with trigger fingers reported unsatisfactory outcomes after surgical treatment. The researchers suggested that the pathology of PIP joint pain may be related to a long duration of symptoms and the consequent joint pathology, including synovitis, joint contracture, or arthrosis [3]. The tenderness of the PIP joint could be a spectrum of coexisting PIP joint conditions with trigger fingers, but this hypothesis has received little attention. There are limited reports on the prevalence and postoperative outcomes of dorsal PIP joint tenderness in patients with trigger fingers.

This study had 2 objectives. The first was to determine the prevalence and risk factors for tenderness of the PIP joint in trigger fingers. The second objective was to compare the postoperative outcomes for the trigger fingers with and without dorsal PIP joint tenderness in terms of postoperative pain, hand function, and range of motion (ROM).

## Methods

### Study design and population

After receiving institutional review board approval, this prospective cohort study was conducted between August 2018 and March 2020. Patients were enrolled if they were 18 years or older, diagnosed with idiopathic trigger finger, and scheduled for open A1 pulley release. All eligible patients consented to participate.

Exclusion criteria were revision surgery, inflammatory joint disease, congenital trigger finger, finger deformity or fixed flexion contracture not consistent with trigger finger, history of fracture or tendon injury, and evidence of osteoarthritic changes or deformity in the affected finger on plain radiography. Patients were also excluded if they received simultaneous additional surgery on the ipsilateral hand (i.e., carpal tunnel release), had a known allergy to paracetamol or codeine (postoperative pain rescue protocol), or developed a surgical wound infection. We estimated the sample size requirements for detecting the effect size of different of the primary outcomes (pain at postoperative 2 weeks) between two groups by using the G*power program (G*Power version 3.1.9.7). The expected effect size was set at 0.5 with 90% statistical power with a significance level of 0.05. With an expected 10% drop-out rate, a total of 190 patients (95 patients per group) were required, however, 89 patients with PIP tenderness were enrolled in this study.

PIP joint tenderness was assessed by pressing on the dorsum of the PIP joint of the affected finger and comparing it with the response of the contralateral finger. “PIP joint tenderness” was defined as pain or discomfort compared to the unaffected finger. Synovitis of the PIP joint was assessed preoperatively using ultrasonography. It was performed by a musculoskeletal radiologist on 52 patients with tenderness of the PIP joint.

### Demographic data

The patient data collected were age, sex, diabetes mellitus status, occupation, hand dominance, duration of the disease, and history of corticosteroid use. In addition, the swelling and flexion contracture of the affected finger were evaluated. Preoperative classification of triggering was used for grading into 4 categories. They were grade I (“pre-triggering”: pain but no catching); grade II (“active correctable”: obvious catching but able to extend the respective digit; grade III (“passive correctable”: overt locking with passive extension or an inability to actively flex; and grade IV (“fixed contracture”: obvious catching with a fixed flexion contraction of the PIP joint) [4].

### Numeric pain scale

Patients were asked to evaluate the extent of pain using an 11-point numeric pain scale (NPS). Ratings ranged from 0 (“no pain whatsoever”) to 10 (“the most intolerable pain”). Various pain measurements of the affected finger were evaluated: pain at rest, pain during finger motion, pain in the A1 pulley, pain at the surgical wound, and pain in the PIP joint.

### ROM

The ROMs of the metacarpophalangeal and PIP joints of the involved finger were measured with a finger goniometer. This instrument is designed to measure the arc or ROM of a particular joint.

### Patient-specific functional scale

The patient-specific functional scale (PSFS) requires patients to choose 3 daily activities they find difficult to perform or for which they are unable to complete a particular movement of the affected hand. Patients were asked to rate the difficulty on a scale from 0 to 10. A score of 0 represented an inability (or much difficulty) to perform the simple daily task, whereas a score of 10 signified ease of motion or no difficulty. The scores for the 3 activities were then summed and averaged for each patient [5].

Postoperative NPS scores, ROM, and PSFS scores were obtained at 1-week, 2-week, and 6-week follow-ups. At 3 and 6 months, the NPS and PSFS scores were obtained by telephone. All examinations and measurements were collected and assessed by a single trained technician.

### Surgical details

The patients were treated with open A1 pulley release under local anesthesia with 1% lidocaine. Operations were performed by any one of several orthopedic surgeons at the study center using the same technique. The A1 pulley was completely transected by a transverse or oblique skin incision, after which the intraoperative active finger flexion was evaluated. Postoperative pain was managed with paracetamol with codeine (300 mg of paracetamol and 15 mg of codeine), and the amount required was recorded. Any additional analgesics that the patients needed were also noted. Suture removal was performed 2 weeks after surgery in most cases.

### Statistical analysis

Demographic and clinical data were summarized with descriptive statistics and compared. Continuous data were assessed for normality with the Kolmogorov–Smirnov test. Normally distributed continuous variables were presented as means and 95% confidence interval, and an independent *t-test* was used to determine the difference between groups. Categorical variables were presented as frequencies and percentages and compared by the chi-squared test or the Fisher exact test. Univariate and multivariate logistic regression analyses were created to determine factors associated with PIP tenderness. Repeated measurements (NPS scores, PSFS, and ROM) were analyzed using a linear mixed model, including all pre- and postoperative time points. Time was included as a repeated effect with an unconstructed covariance structure. Between groups (with or without PIP tenderness), time and the interaction group × time were set as fixed effects. The *p*-values < 0.05 were considered statistically significant. Due to some possible violation of normality assumptions, the Mann–Whitney test was used for sensitivity analysis for the significantly different functional outcomes between groups. However, as no different results were observed, these data are not reported. The *p*-values < 0.05 were considered statistically significant. For repeated measurement, the *p*-value was adjusted with the Bonferroni method by multiplying by the number of tests. All statistical analyses were performed using IBM SPSS Statistics for Windows, Version 23.0 (IBM Corp., Armonk, NY, USA).

## Results

Of the 190 participants, 89 (47%) had dorsal PIP joint tenderness. Table 1 details the demographic and clinical characteristics of the groups with and without dorsal PIP joint tenderness. By using a univariate and multivariate logistic regression analyses to determine factors association with PIP tenderness, a statistically significant difference was observed between occupations and joint swelling. A significantly greater proportion of patients who had PIP joint tenderness and were self-employed or engaged in light manual work exhibited coexisting dorsal PIP joint tenderness (64%) than patients who did not have PIP joint tenderness. However, there were no significant differences between the 2 groups for office workers, homemakers, and retired or unemployed individuals.

Moreover, no statistical differences were observed between the 2 groups regarding age, sex, disease duration, history of corticosteroid use, affected finger, trigger finger grading, or whether the affected finger was on the dominant hand. Diabetes mellitus was equally distributed in both groups. The long and middle fingers were the most affected in both groups (41.6%). PIP joint swelling was found significantly more frequently among patients with PIP joint tenderness (84.3% vs. 63.4%; *P* = 0.002). Among 89 patients with PIP joint tenderness, 52 underwent ultrasound examination to evaluate synovitis of the PIP joint. Only 2 of the 52 patients (3.8%) had a positive sign of synovitis.

**Table 1:** Demographic data

Demographic data	Total (*N* = 190)	
	PIP joint tenderness (*n* = 89)	Without PIP joint tenderness (*n* = 101)
Age (years)	61.1 (SD 8.8)	59.8 (SD 8.5)
Female sex	72 (80.9%)	79 (78.2%)
Diabetes mellitus	20 (22.5%)	20 (19.8%)
Occupation		
- Unemployed/homemaker/retired	41 (46.0%)	66 (65.3%)
- Office worker	28 (31.5%)	24 (23.8%)
- Self-employed/Light manual worker*	20 (22.5%)	11 (10.9%)
Duration of disease (months)	7.4 (SD 7.0)	7.8 (SD 7.8)
Previous corticosteroid injections	40 (46%)	47 (54%)
Disease affected dominant hand	57 (64%)	61 (60.4%)
Involved finger		
- Thumb	16 (18%)	29 (28.7%)
- Index	16 (18%)	12 (11.9%)
- Middle	36 (40.4%)	43 (42.6%)
- Ring	20 (22.5%)	16 (15.8%)
- Small	1 (1.1%)	1 (1.0%)
Trigger finger grading		
- II	26 (29.2%)	41 (40.6%)
- III	33 (37.1%)	35 (34.7%)
- IV	30 (33.7%)	25 (24.8%)
PIP joint swelling*	75 (84.3%)	64 (63.4%)

*SD* standard deviation

*Statistically significant (*P* < 0.05)

**Fig. 1:**
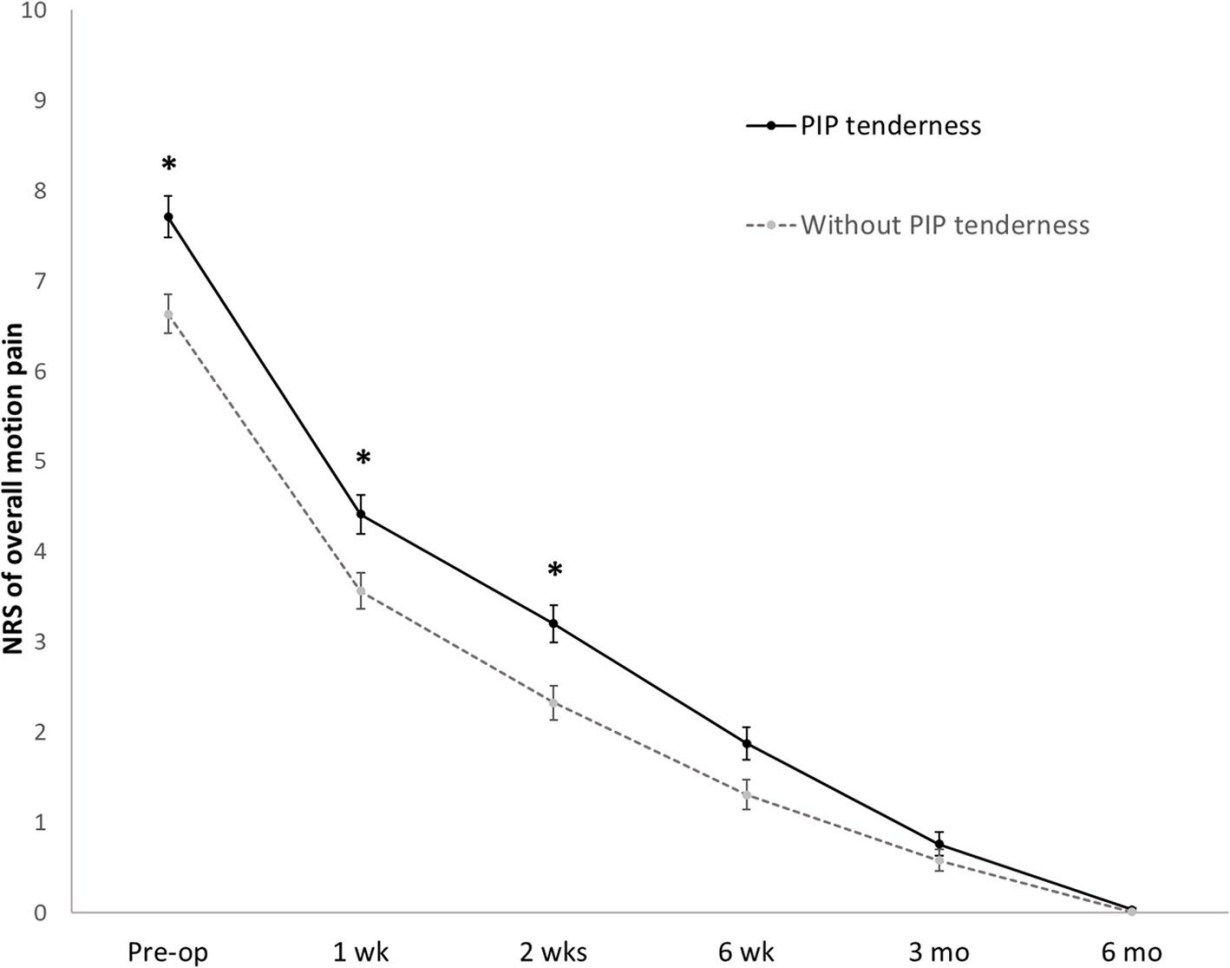
Comparison of pre- and postoperative numeric pain scale scores overall motion digit pain for each group at each follow-up time point using linear mixed-model analyses. Statistically significances of motion pain were observed between groups (*p* < 0.05) at pre- and postoperative 1 week, 2 weeks

The overall pre- and postoperative digit rest pain and digit motion pain are illustrated in Fig. 1. Table 2 presents a detailed comparison of the functional outcomes of the PIP tenderness and without PIP tenderness groups at each follow-up time point. Patients with PIP tenderness reported significantly higher pain scores for rest and motion pain. However, digit motion pain was more affected than rest pain for all patients. The average NPS score of 3.8 at rest increased to 7.7 during digit motion for patients with PIP tenderness, compared with a rise from 2.5 to 6.6 for the group without PIP tenderness.

**Table 2:** Comparison of functional outcomes of PIP tenderness and without PIP tenderness groups at each follow-up time point

Functional outcomes	PIP tenderness (*n* = 89)	Without PIP tenderness (*n* = 101)	Difference Between Groups	*P* value^a^
	Mean (95% CI)	Mean (95% CI)	Mean (95% CI)	
**Overall resting digit pain **^**b**^				
Pre-op	3.8 (3.3–4.3)	2.5 (2.0–3.0)	1.2 (0.5–1.9)	0.001* (0.001)
Post-op 1 week	1.8 (1.5–2.2)	0.9 (0.6–1.2)	0.8 (0.4–1.2)	< 0.001* (< 0.001)
Post-op 2 weeks	1.0 (0.7–1.2)	0.4 (0.2–0.7)	0.4 (0.1–0.6)	0.068 (0.017)
**Overall motion digit pain**				
Pre-op	7.7 (7.3–8.2)	6.6 (6.2–7.0)	1.1 (0.4–1.7)	0.001* (0.001)
Post-op 1 week	4.4 (4.0–4.8)	3.6 (3.2–4.0)	0.8 (0.2–1.4)	0.030* (0.005)
Post-op 2 weeks	3.2 (2.8–3.6)	2.3 (1.9–2.7)	0.9 (0.3–1.4)	0.018*(0.003)
Post-op 6 weeks	1.9 (1.5–2.2)	1.3 (1.0–1.6)	0.5 (0.1–1.0)	0.138 (0.023)
Post-op 3 months	0.8 (0.5–1.0)	0.6 (0.3–0.8)	0.2 (0.2–0.5)	1.000 (0.354)
**PSFS**				
Pre-op	5.5 (5.0–5.9)	5.3 (4.9–5.7)	0.2 (-0.4–0.8)	1.000 (0.542)
Post-op 1 week	5.5 (4.9–6.1)	6.1 (5.6–6.7)	-0.6 (-1.4–0.1)	0.660 (0.110)
Post-op 2 weeks	7.3 (6.9–7.7)	7.6 (7.2–8.0)	-0.3 (-0.9–0.3)	1.000 (0.282)
Post-op 6 weeks	8.5 (8.3–8.8)	8.8 (8.5–9.1)	-0.3 (-0.6–0.1)	1.000 (0.192)
Post-op 3 months	9.4 (9.2–9.6)	9.5 (9.4–9.7)	-0.1 (-0.3–0.1)	1.000 (0.282)
**TAM (degrees)**				
Pre-op	187.3 (175.9–198.7)	175.5 (175.9–198.7)	13.4 (-1.5–28.3)	0.308 (0.077)
Post-op 1 week	173.1 (163.7–182.4)	170.1 (160.8–179.4)	2.6 (-7.6–12.9)	1.000 (0.612)
Post-op 2 weeks	198.6 (190.7–206.5)	200.2 (192.3–208.0)	-1.9 (-10.4–6.6)	1.000 (0.658)
Post-op 6 weeks	221.9 (214.0–229.8)	221.5 (213.2–229.9)	0.4 (-11.1–11.8)	1.000 (0.954)
**PIP extension (degrees)**^**c**^				
Pre-op	-1.2 (-3.6–0.9)	0.5 (-1.5–2.5)	-0.1 (-2.7–2.6)	1.000 (0.964)
Post-op 1 week	-1.1 (-3.2–1.0)	0.4 (-1.5–2.3)	0.2 (-1.8–2.3)	1.000 (0.859)
Post-op 2 weeks	0.5 (-1.5–2.5)	3.9 (2.0–5.8)	-1.7 (-3.4–0.1)	0.060 (0.240)
Post-op 6 weeks	4.1 (2.0–6.2)	5.8 (3.7–7.9)	-1.6 (-4.7–1.3)	1.000 (0.259)

Data derived from linear mixed model analysis for repeated measurements; ^a^ The *p* values adjusted by Bonferroni analysis are presented, with the *p* values before the Bonferroni analysis in parentheses (6 comparisons for motion pain and PSFS and 4 comparisons for rest pain, TAM and PIP extension); *Statistically significant (*P* < 0.05); ^b^ Overall digit pain at rest subsided by 6 weeks follow-up; ^c^ extension (-), flexion contracture; extension (+), hyperextension; PSFS, patient-specific functional scale; TAM, total arc of motion

After the A1 pulley was released, overall digit rest pain was significantly higher for patients with PIP tenderness, but only during the early postoperative weeks. However, in the first postoperative week, while patients without PIP tenderness had abruptly decreased rest pain, with NPS scores ranging from 2.5 to 0.9, patients with PIP tenderness still experienced some pain (mean NPS score = 1.8). At the second postoperative week, although there was a statistically significant higher rest pain level for the PIP tenderness group, the pain levels for both groups were subtle (mean NPS score of both groups < 1).

The overall motion pain significantly decreased for both groups during the postoperative period, although with a substantially higher pain score for the PIP tenderness group during the initial 2-week follow-up period (*P* < 0.05). The PSFS score, total arc of motion, and PIP extension also improved gradually over the follow-up period, but without statistical difference between the patients with or without PIP tenderness. The postoperative range of motion was not affected by coexisting PIP tenderness. However, patients with PIP tenderness had a slight loss of PIP extension and lower PSFS score at 2 weeks after surgery.

The time, between-group, and interaction effects on functional outcomes were analyzed using a linear mixed model and are summarized in Table 3. Pain scores, PSFS scores, and motion improved substantially during the follow-up period (*P* < 0.05). However, only overall digit pain revealed a significant difference between groups and found an interaction between time and PIP tenderness (*P* < 0.05). At the 6-week follow-up, the mean motion pain score for the PIP tenderness group was 1.9 compared with 1.3 for patients without PIP tenderness, it might not be clinically significant. However, we found that 16 patients (18%) with PIP tenderness reported persistently significant pain (mean NPS score > 3) during digit motion, compared with only approximately 8% of the patient without PIP tenderness (18% vs. 7.9%; P = 0.04). Although almost all pain had subsided by 3 months postoperatively in both groups, approximately half of the patients with PIP tenderness (48.3%) and only one-third of the patients without PIP tenderness complained of residual minor pain on digit motion (48.3% vs. 33.7%; *P* = 0.04).

**Table 3:** Comparison of linear mixed model results of the effect of time on functional outcomes for the PIP tenderness and without PIP tenderness groups

Functional outcomes	Time effect		Between-group effect		Interaction effect	
	F test	*P* value	F test	*P* value	F test	*P* value
Overall resting digit pain	96.8	< 0.001*	19.1	< 0.001*	6.2	< 0.001*
Overall motion digit pain	461.65	< 0.001*	13.32	< 0.001*	3.65	0.01*
PSFS	199.8	< 0.001*	1.12	0.292	0.97	0.44
TAM	141.06	< 0.001*	0.51	0.48	1.59	0.19
PIP extension	36.49	< 0.001*	2.60	0.11	2.56	0.06

*PSFS* patient-specific functional scale, *TAM* total arc of motion

* Statistically significant (*P* < 0.05)

## Discussion

Pain and stiffness around the PIP joint are among the most significant clinical findings in trigger finger pathology. In a 2018 study, Kim and coauthors reported that 24% of 179 patients exhibited PIP joint pain concurrently with prolonged symptoms of trigger finger, fixed extension loss, and joint tenderness. Postoperatively, these patients had higher pain scores and considerable dissatisfaction with the recovery process at a mean follow-up of 18 months. However, the etiology of the PIP joint pain was unknown [3]. Possible causes may be overuse of the affected digit, synovitis (inflammation), or early degenerative processes of the PIP joint; the latter two are undetectable by plain radiography or flexor tendon pathology. In contrast to PIP joint pain, dorsal PIP joint tenderness was not described in earlier reports.

The current work focused on dorsal PIP tenderness and found that 46.8% of patients with trigger fingers had coexisting dorsal PIP joint tenderness. Unlike the report by Kim et al., we did not find a correlation between tenderness and either symptom duration or fixed extension loss. The mean duration of the symptoms found by Kim and associates was approximately 23 months. This period was markedly longer than that of our study (8 months), and it may be one of the explanations for the different results. In addition, the research by Kim and colleagues investigated PIP joint pain. In comparison, our study examined dorsal PIP tenderness, which accounted for only 13% of their patients [3].

The patient demographic data associated with PIP joint tenderness were not impressive in terms of severity, duration of symptoms, age, sex, or diabetes. The only exception was the occupation variable. Self-employed or light manual workers more frequently had coexisting PIP tenderness than homemakers, office workers, and retired or unemployed patients.

We found that dorsal PIP joint tenderness was associated with poorer surgical outcomes for the trigger fingers. Patients with tenderness had higher rest and motion pain scores before surgery and for up to 6 weeks after the procedure. Additionally, more patients with PIP tenderness still had pain scores > 3 at the 6-week follow-up and reported residual minor pain on digit motion for up to 3 months after the operation.

Coexisting joint pathology may be associated with poorer postoperative results due to another risk factor [6, 7]. For example, diabetes mellitus can cause residual stiffness or other postoperative complications. Diabetic patients were not excluded and were equally distributed in both study groups. Therefore, the comparative results were not affected.

The pathogenesis of dorsal PIP tenderness in patients with trigger fingers has not yet been established. Synovitis of the PIP joint might not be a significant cause as only 2 of the 52 patients with PIP tenderness had a positive sign of synovitis detected by ultrasonography. The ultrasonographic features of trigger fingers demonstrated by Chuang et al. [8] suggested that triggering occurs when the enlarged flexor tendon, especially the flexor digitorum superficialis bifurcation, moves underneath the thickened A1 pulley while flexing the finger. These findings support the previous results of a biomechanical study by Lu et al [9]. Their kinematic and kinetic study of trigger fingers revealed that the force of flexors that required extensors to overcome the catching was 1.54 times greater than that of healthy fingers [9]. Therefore, we propose that the imbalance between mechanical strain and abnormal anatomical architectures in trigger fingers leads to inflammation and, in turn, tenderness and swelling of the dorsal PIP. However, further investigations are needed to prove this hypothesis.

Many other confounders can affect postoperative outcomes. For example, Cakmak and colleagues found that light manual workers had significantly longer postoperative symptoms. Patients with preoperative systemic steroids and trigger thumbs recovered faster [10]. Baek et al. reported that prolonged postoperative symptoms were associated with a longer duration of disease, preoperative flexion contracture, and flexor tendon injury [11]. PIP joint pain was also a risk factor for poorer postoperative satisfaction, as indicated earlier [3]; however, PIP joint tenderness has not previously been described as a factor affecting outcomes.

The current study controlled specific factors to identify the influence of coexisting dorsal PIP tenderness on surgical outcomes. They were age, sex, associated diseases, disease duration, severity, and use of steroids and nonsteroidal anti-inflammatory drugs. The surgical operations in the study were conducted in the same surgical suite, and the surgeons used the same technique throughout the study to avoid potential confounder effects related to the operation.

A possible limitation of this study was that the same surgeon did not perform the surgical intervention. However, all patients were assigned to the same surgical suite, so it can be assumed that the operative technique of open A1 pulley release was similar in all cases.

## Conclusion

Dorsal PIP tenderness is more common in trigger fingers than previously thought. It is also associated with higher and prolonged postoperative pain after A1 pulley release. Therefore, patients with pre-existing PIP tenderness should be informed about the possibility of sustaining residual minor pain for up to 3 months after surgery.

## Data Availability

The datasets used or analyzed during the current study are available from the corresponding author on reasonable request.
